# Sonographers' self‐reported visualization of normal postmenopausal ovaries on transvaginal ultrasound is not reliable: results of expert review of archived images from UKCTOCS

**DOI:** 10.1002/uog.18836

**Published:** 2018-03-07

**Authors:** W. Stott, S. Campbell, A. Franchini, O. Blyuss, A. Zaikin, A. Ryan, C. Jones, A. Gentry‐Maharaj, G. Fletcher, J. Kalsi, S. Skates, M. Parmar, N. Amso, I. Jacobs, U. Menon

**Affiliations:** ^1^ Women's Cancer UCL EGA Institute for Women's Health London UK; ^2^ Create Health Clinic London UK; ^3^ London School of Hygiene and Tropical Medicine London UK; ^4^ Biostatistics Center Massachusetts General Hospital Boston MA USA; ^5^ Medical Research Council Clinical Trials Unit at UCL London UK; ^6^ School of Medicine, College of Biomedical and Life Sciences Cardiff University Cardiff UK; ^7^ University of New South Wales, Sydney Australia

**Keywords:** automated image analysis, expert review, ovarian cancer screening, quality control, transvaginal sonography, UKCTOCS, ultrasound, visualization rate

## Abstract

**Objective:**

In the UK Collaborative Trial of Ovarian Cancer Screening (UKCTOCS), self‐reported visualization rate (VR) of the ovaries by the sonographer on annual transvaginal sonographic (TVS) examinations was a key quality control (QC) metric. The objective of this study was to assess self‐reported VR using expert review of a random sample of archived images of TVS examinations from UKCTOCS, and then to develop software for measuring VR automatically.

**Methods:**

A single expert reviewed images archived from 1000 TVS examinations selected randomly from 68 931 TVS scans performed in UKCTOCS between 2008 and 2011 with ovaries reported as ‘seen and normal’. Software was developed to identify the exact images used by the sonographer to measure the ovaries. This was achieved by measuring caliper dimensions in the image and matching them to those recorded by the sonographer. A logistic regression classifier to determine visualization was trained and validated using ovarian dimensions and visualization data reported by the expert.

**Results:**

The expert reviewer confirmed visualization of both ovaries (VR‐Both) in 50.2% (502/1000) of the examinations. The software identified the measurement image in 534 exams, which were split 2:1:1 providing training, validation and test data. Classifier mean accuracy on validation data was 70.9% (95% CI, 70.0–71.8%). Analysis of test data (133 exams) provided a sensitivity of 90.5% (95% CI, 80.9–95.8%) and specificity of 47.5% (95% CI, 34.5–60.8%) in detecting expert confirmed visualization of both ovaries.

**Conclusions:**

Our results suggest that, in a significant proportion of TVS annual screens, the sonographers may have mistaken other structures for normal ovaries. It is uncertain whether or not this affected the sensitivity and stage at detection of ovarian cancer in the ultrasound arm of UKCTOCS, but we conclude that QC metrics based on self‐reported visualization of normal ovaries are unreliable. The classifier shows some potential for addressing this problem, though further research is needed. © 2017 The Authors. *Ultrasound in Obstetrics & Gynecology* published by John Wiley & Sons Ltd on behalf of the International Society of Ultrasound in Obstetrics and Gynecology.

## INTRODUCTION

Transvaginal sonography (TVS) is used widely for pelvic imaging, in the context of both patient management and ovarian cancer screening. Visualization of the ovaries is a desired prerequisite but can be challenging in older women as their ovaries are typically shrunken or difficult to locate.

Visualization rate (VR), the percentage of all exams performed by the sonographer in which the ovaries are identified, is a quality control (QC) metric used widely in TVS scanning for ovarian cancer screening^1^. However, there is variation across different studies with respect to how VR is defined, with some reporting visualization of both ovaries (VR‐Both) and some of one or both ovaries (Table 1)^1^, ^2^, ^3^, ^4^, ^5^, ^6^, ^7^, ^8^. We believe that in the context of ovarian cancer screening, VR‐Both is a more meaningful metric as early cancer can begin in one ovary before spreading to the contralateral one.

**Table 1:** Variation in visualization rate (VR) in studies of transvaginal ultrasound examination of ovaries, according to different definitions for visualization

Trial/study	Examinations (*n*)	Dates	Definition of VR	Reported VR (%)
UKCTOCS^2^	270 035	June 2001–Dec 2011	RO or both	72.7
			One or both	84.5
UKCTOCS^1^	43 867	June 2001–Aug 2007	RO or both	66.8
			LO or both	65.5
PLCO^3^	102 787	1993–2009	Both	60
Kentucky^4^	57 214	1987–1999	One or both	79.2
Kentucky^5^	120 569	1987–2005	One or both	84
Kentucky^6^	205 190	1987–2011	One or both	87.6
Ludovisi (2014)^7^	6649	Oct 2008–Sept 2013	RO or both	84.1
			LO or both	82.4
Gollub (1993)^8^	206	June 1988–Mar 1989	Both	49
			One or both	80

Only first author is given for last two studies.

Kentucky, Kentucky ovarian cancer ultrasound screening study; LO, left ovary; PLCO, Prostate, Lung, Colorectal, and Ovarian cancer screening trial; RO, right ovary; UKCTOCS, UK Collaborative Trial of Ovarian Cancer Screening.

Obtaining reliable VR data is challenging as ovarian visualization is subjective and sensitive to inter‐ and intraobserver variation^9^. In addition, all previous studies^1^, ^2^, ^3^, ^4^, ^5^, ^6^, ^7^, ^8^ have calculated ovarian VR using visualization data self‐reported by the sonographers. In the UK Collaborative Trial of Ovarian Cancer Screening (UKCTOCS), self‐reported VR was the QC metric used during annual ultrasound screening for ovarian cancer. Static ultrasound images obtained at the time of the examination were archived centrally, providing an opportunity to investigate retrospectively whether ovarian visualization had been achieved successfully. We are not aware of any previous study that has attempted such a TVS validation apart from an audit of seven sonographers reporting high VR performed by our group (Stott *et al*., in prep.).

In the current study, we report the results of a retrospective expert review of a random sample of archived static images from annual TVS examinations performed in UKCTOCS, which were classified as normal and performed between 2008 and 2011, after introduction of scanning accreditation and improvement of quality monitoring^2^. Our study also attempts to address the challenge of obtaining an objective measure of VR, unaffected by inter‐/intraobserver variability, by constructing a software classifier trained using data from the expert review, with the aim of supporting future quality improvement in TVS.

## METHODS

UKCTOCS was a multicenter randomized controlled trial involving 202 638 female volunteers from 13 trial centers in England, Wales and Northern Ireland. Inclusion criteria were postmenopausal women aged 50–74 years at recruitment. Women with previous ovarian malignancy, bilateral oophorectomy, active non‐ovarian malignancy or increased risk of familial ovarian cancer, and those participating in other ovarian cancer screening trials were excluded. The women were randomized into three groups: (1) ultrasound screening (*n* = 50 639), (2) multimodal screening using CA 125 interpreted by the Risk of Ovarian Cancer (ROC) algorithm (*n* = 50 640) and (3) no screening (control; *n* = 101 359)^10^. Women in the ultrasound group underwent annual screening using TVS or transabdominal sonography (TAS) when TVS was not acceptable to the volunteer. Details of the ultrasound screening process and its reporting have been described previously^2^. An important part of the examination results was capturing the dimensions of each ovary in two orthogonal planes, which allowed calculation of the ovarian volume. Annual screening in the ultrasound assessment arm of UKCTOCS occurred between 4 July 2001 and 21 December 2011. A total of 328 867 annual ultrasound scans were performed on 48 250 volunteers, of which 300 027 were performed by TVS.

**Figure 1:**
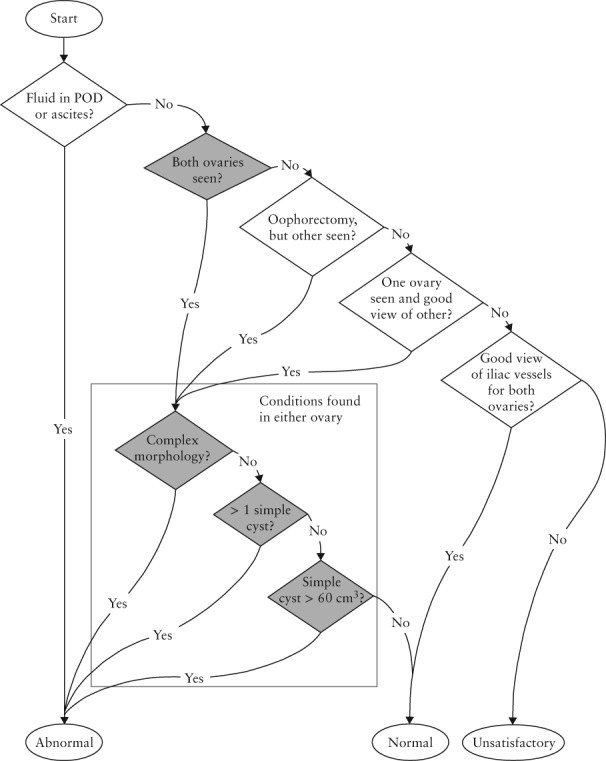
UK Collaborative Trial of Ovarian Cancer Screening (UKCTOCS) algorithm for classification of pelvic scans based on data reported by sonographer. ‘Ovary seen’ indicates that ovary is visualized; ‘good view’ indicates that ovary is not seen but > 4 cm of iliac vessels are seen. POD, pouch of Douglas.

A bespoke Trial Management System (TMS) implemented the algorithm described in the trial protocol for categorizing the TVS examinations as abnormal, unsatisfactory or normal, based on data reported by the sonographer for each ovary (Figure 1). These data included measurements (D1, D2, D3) of the left and right ovaries copied from values displayed by the ultrasound machine after the sonographer had placed caliper marks on the boundaries of the ovary in a static image captured for this purpose. A further bespoke computer system, the Ultrasound Record Archive (URA), was developed to archive these static images, as reported elsewhere^11^.

The images from 216 152 TVS examinations (72% of all TVS annual scans performed in UKCTOCS) were archived in the URA, including 113 093 scans that were performed after January 2008 when quality monitoring had been improved, scanning accreditation had been introduced and the ultrasound machines at all 13 trial centers were upgraded to the Accuvix XQ model (Medison, Seoul, South Korea)^2^. These later examinations were performed by 141 sonographers all accredited to perform annual (level 1) TVS scans^1^.

The archived images from 1000 ultrasound examinations classified as normal by the sonographers were selected randomly for inclusion in the study dataset. Inclusion criteria were: (1) annual TVS examination of women in the ultrasound screening group; (2) images stored in the URA; (3) performed after 1 January 2008; (4) both ovaries measured/visualized; and (5) both ovaries categorized as having normal morphology. Examinations performed by TAS only and those categorized as abnormal or unsatisfactory were excluded.

The UKCTOCS study was approved by the North West Multicentre Research Ethics Committee (21/6/2000; MREC reference 00/8/34) and it is registered as an International Standard Randomised Controlled Trial (no. ISRCTN22488978).

### Expert review

Images associated with each of the 1000 scans in the dataset were copied from the URA as 640 × 480 pixel grayscale bitmap files. A spreadsheet containing hyperlinks to these images was prepared so the reviewer could display them by selecting the appropriate cell, as reported elsewhere^11^. Bespoke software was used to process the images in order to measure the caliper marks. The resultant dimensions were matched against the ovary dimensions recorded in the TMS in order to identify the exact image the sonographer had used to measure the ovary^11^. The spreadsheet was annotated to indicate the images that had been used to measure the ovaries. However, the expert reviewed all images of each examination in the dataset to evaluate any bias arising from software selection.

**Figure 2:**
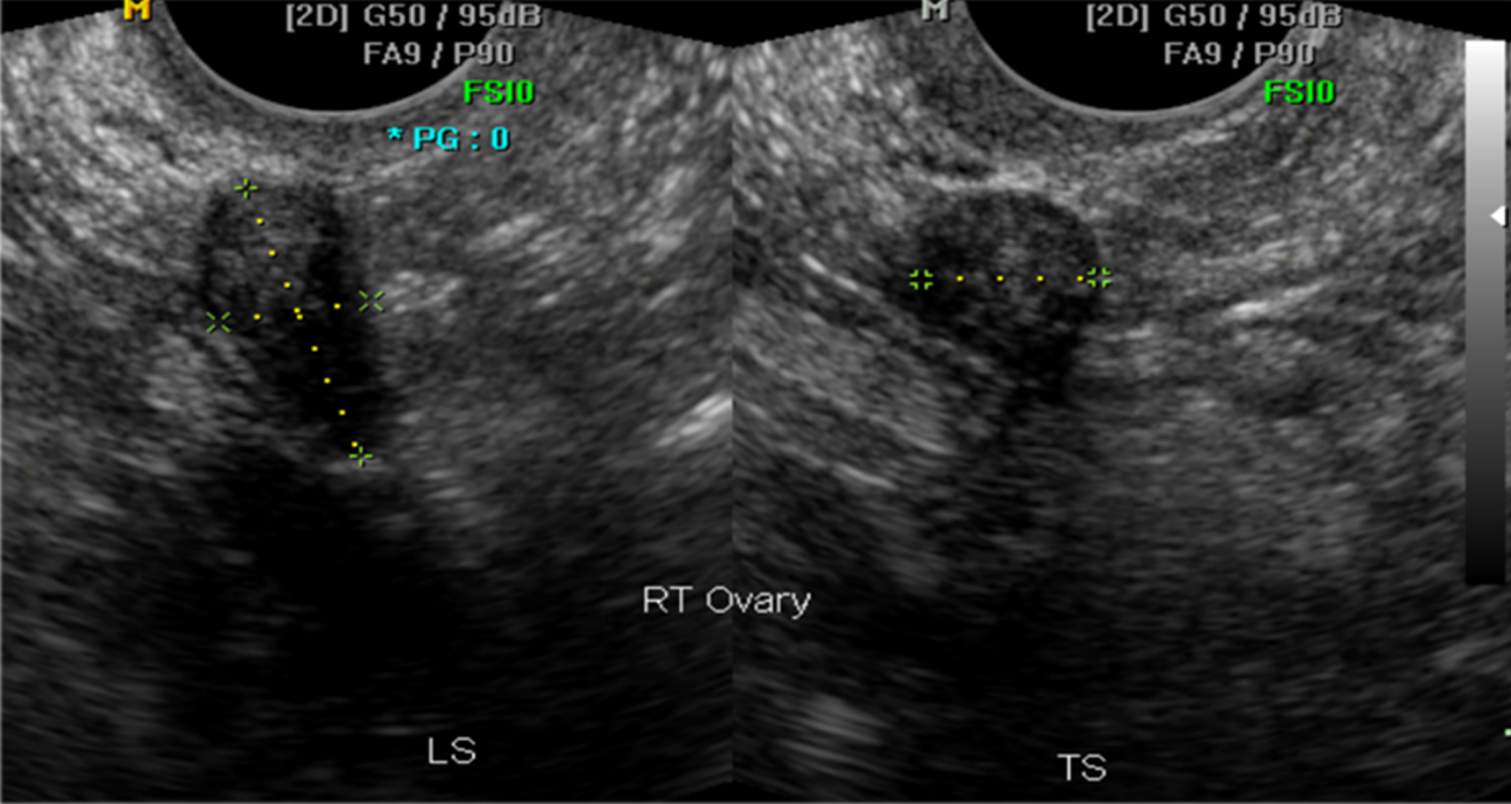
Longitudinal (LS) and transverse (TS) transvaginal ultrasound images of right ovary acquired by sonographer. This ovary was confirmed by the expert reviewer as normal and measured correctly.

**Figure 3:**
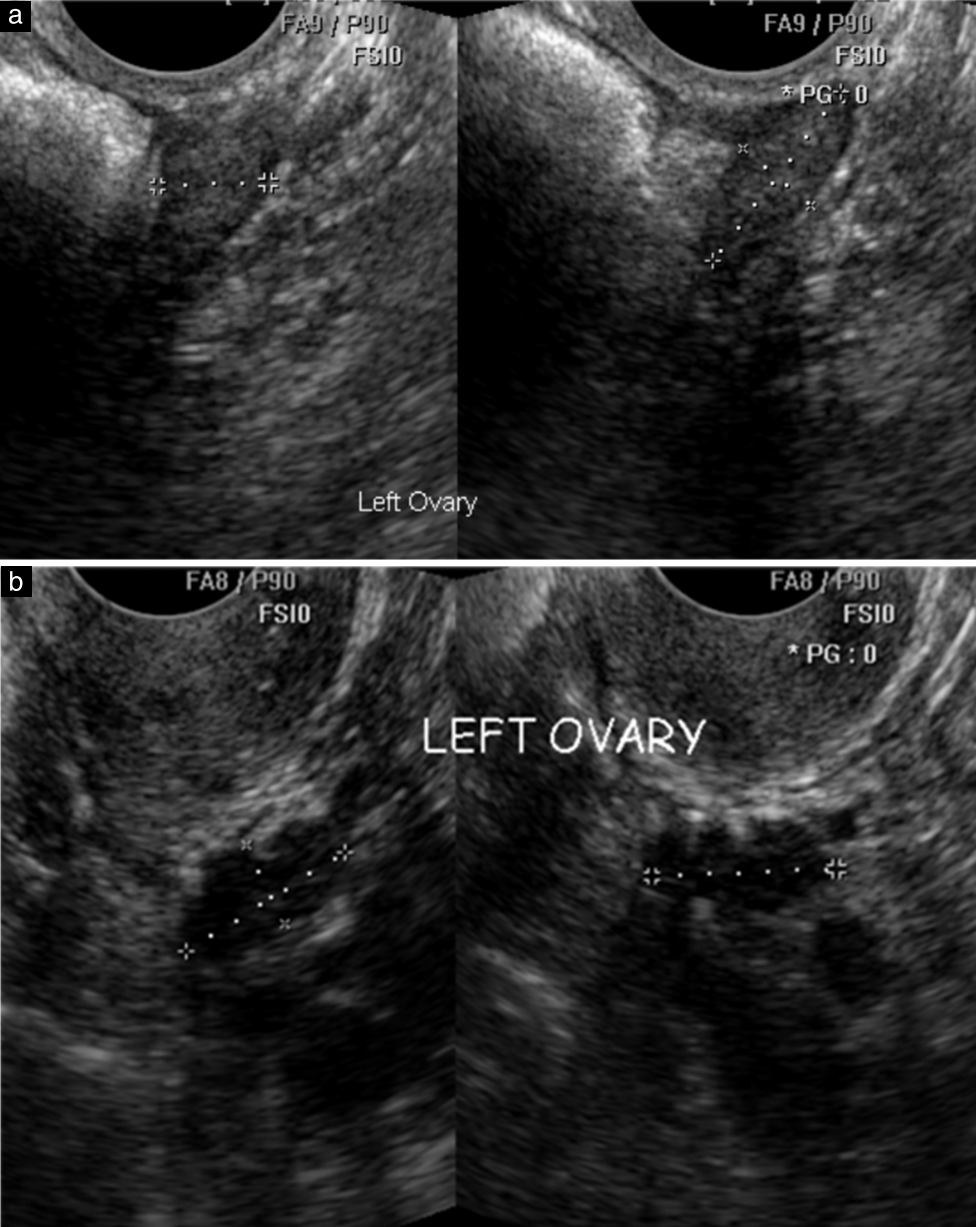
Longitudinal and transverse transvaginal ultrasound images acquired by sonographers to measure left ovaries in postmenopausal women. (a) The expert judged the ovary as normal and correctly measured by the sonographer. (b) The expert considered the sonographer had mistakenly measured a section of bowel rather than the ovary as the haustrations of large bowel are clearly visible in the structure marked by the calipers.

A single expert in gynecological scanning reviewed the images of each examination and recorded assessments of the left and right ovaries using one of the following categorical variables: visualized and correctly measured; visualized but poorly measured; not visualized; and not appropriate (images not of the adnexal region, such as of the uterus). Criteria used to indicate that an ovary was not visualized were an irregular or indistinct outline, a heterogeneous echogenicity of the stroma and an outline that could be identified as part of a larger shape, which was usually the bowel (Figures 2 and 3). In practical terms, the expert mentally removed the calipers and if the resulting shape did not resemble an ovary then the image was classified as ‘not visualized’. This was confirmed occasionally by measurements that were clearly outside the normal range expected for a postmenopausal ovary.

### Construction of logistic regression classifier

Statistical learning techniques were used to construct a logistic regression classifier using R version 3.3.2 (R Foundation for Statistical Computing, Vienna, Austria). It used the train function provided by the CARET (Classification and REgression Training) package version 6.0.71 with the generalized linear model (glm) specified as its method parameter. Ovarian dimensions and ovary side (left or right) were used as candidate feature data. Ovary visualization (true or false, as judged by the expert reviewer) was used as the target value.

VRs were calculated from the results of the expert review of all the images associated with the 1000 examinations included in the dataset. An ovary was defined as ‘seen’ when the expert reviewer categorized the image using the categorical variables ‘visualized and correctly measured’ or ‘visualized but poorly measured’. The use of any other categorical variable was defined as ‘not seen’. VRs were calculated for all 1000 examinations in the dataset using various VR definitions (Table 2).

**Table 2:** Visualization rate (VR) of ovaries on expert review according to definition of VR used

Expert VR definition	Exams with ovarian images identified by software		Exams with ovarian images not identified by software		All exams	
	Count (n = 534)	Expert VR (%)	Count (n = 466)	Expert VR (%)	Count (n = 1000)	Expert VR (%)
RO or both (right ovary or both ovaries seen)	366	68.5	297	64.0	663	66.3
LO or both (left ovary or both ovaries seen)	344	64.4	286	61.4	630	63.0
One or both (left or right ovary seen or both ovaries seen)	430	80.5	362	77.7	792	79.2
Both (both ovaries seen)	280	52.4	222	47.6	502	50.2

LO, left ovary; RO, right ovary.

The images in the dataset were processed to create two subsets: (1) the ‘match’ subset containing examinations for which the software found the exact images used by the sonographer to measure the left and right ovaries by matching the caliper measurements calculated by the software against those reported by the sonographer, and (2) the ‘no match’ subset containing examinations for which the software could not find the exact images used by the sonographer to measure the ovaries. To facilitate analysis, examinations that did not have the longitudinal and transverse sections of the ovary in the same image for both left and right ovaries were excluded. Visualization rates (VR) were calculated for the exams in both subsets using the definitions in Table 2 so that the differences between them could be assessed.

The ‘match’ subset was randomly split in the ratio 2: 1: 1 (training, validation, test) in order to build the logistic regression classifier. Various combinations of features were used to construct models from the same selection of training and validation data so the performance of each could be evaluated in terms of accuracy. The combination of features that offered best performance was selected and the data were split randomly using different seed values so that performance could be measured for different (same sized) collections of training and validation data as randomly selected. Mean value with 95% CI for accuracy (mean true positives plus mean true negatives divided by total observations for ovary dimensions in the randomly selected validation data), sensitivity and specificity were calculated. Mean values for true positive, true negative, false positive and false negative results (as defined in Table 3) were obtained by averaging the values obtained for each selection of exams used as validation data, split randomly from the ‘match’ data subset. In this way the classifier performance metrics were not dependent on any particular selection of exams from the ‘match’ data subset.

**Table 3:** Contingency table comparing visualization of both ovaries by classifier with that on expert review in test dataset of 133 examinations

Visualization by classifier	Visualization on expert review	
	Both ovaries visualized	One or both ovaries not visualized
Both ovaries visualized	67 (TP)	31 (FP)
One or both ovaries not visualized	7 (FN)	28 (TN)

FN, false negatives; FP, false positives; TN, true negatives; TP, true positives.

The combination of features with the best performance was taken from exams in the test data and applied to the classifier. The classifier generated a result for each image in terms of ovary visualized, or not. These results were then used to determine visualization of the ovaries for each examination and the values were used to calculate confirmed VR‐Both (cVR‐Both) for the test data.

## RESULTS

A total of 113 093 annual TVS examinations with images archived in the URA were performed after 1 January 2008. The TMS categorized these examinations according to the trial protocol (Figure 1) yielding 105 176 (93%) normal scans, 5097 (4.5%) abnormal scans and 2820 (2.5%) unsatisfactory scans. The dataset of 1000 examinations was selected randomly from 68 931 of the 105 176 normal examinations in which both ovaries were reported as ‘seen’ (visualized) and measured. This dataset included a total of 4654 images with a mean of 4.6 images per exam (range, 1–15).

The results of the assessment of the images by the expert reviewer allowed calculation of VR, but the values changed significantly depending on the definition used for visualization. Using a definition of both ovaries confirmed as visualized (cVR‐Both) the value of VR was 50.2%, but the value changed to 79.2% when a definition of one or both ovaries was applied (Table 2).

The software was configured to identify exams in which both ovaries had their transverse and longitudinal sections recorded in the same image and in which the software caliper measurements could be matched to those reported by the sonographer. A match subset of 534 (53.4%) such examinations was created. These were examinations for which the software had found the exact images used by the sonographer to measure the ovaries. The images used by the sonographer to measure the ovaries were identified in a further 17 (1.7%) exams but were excluded as the transverse and longitudinal sections were not in the same image. In the remaining 449 (44.9%) exams, the images used by the sonographer to measure the ovaries could not be identified for the following reasons: duplicate images (8.6%), unresolved (16.4%), process failure (3.9%), non‐standard caliper marks in the images (16.0%)^11^. The cVR‐Both results based on expert review of the images in the ‘match’ subset of 534 examinations were not significantly different from those of images in the ‘no‐match’ subset of the remaining 466 exams (Table S1).

**Figure 4:**
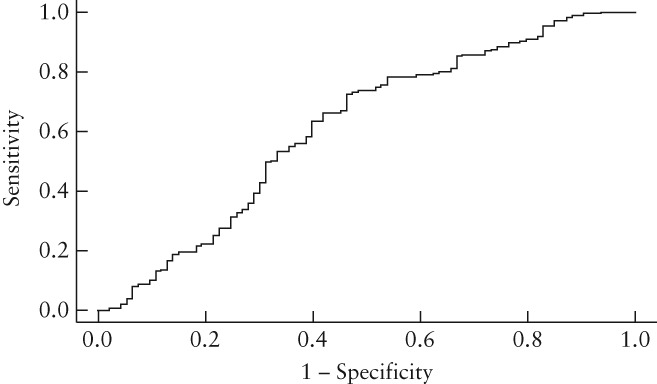
Receiver–operating characteristics curve showing performance of classifier for individual ovaries using validation data values from random split of the 534 examinations in ‘match’ data subset into training, validation and test data.

The performance of the classifier was evaluated using the validation data comprising 268 of 1068 ovary dimensions in the ‘match’ subset of 534 examinations. Thirty different collections of validation data were generated by splitting randomly the subset using different seed values, each having feature and target data from the left and right ovaries in 134 exams, thus 268 in total. The results of each collection were calculated as described in ‘Methods’. Mean accuracy was 70.9% (95% CI, 70.0–71.8%), mean sensitivity was 93.0% (95% CI, 92.1–94.0%) and mean specificity was 27.3% (95% CI, 25.4–29.3%). A receiver–operating characteristics curve (Figure 4) was produced from validation data in the same random split of the ‘match’ subset data that contained the test data used to calculate cVR‐Both.

The test data were formed by 266 ovarian dimensions from 133 exams in the ‘match’ data subset that had not been used for training or validation. Forty‐seven sonographers performed these exams, with a mean of 2.83 (range, 1–15) exams performed by individual sonographers. When the test data were applied to the classifier, cVR‐Both was 73.7% compared with the gold standard of 55.6% found by the expert. The expected accuracy was calculated as 61.8%, which gave a kappa value of 0.24 (judged only fair according to the Landis–Koch interpretation)^12^ with sensitivity of 90.5% (95% CI, 80.9–95.8%) and specificity of 47.5% (95% CI, 34.5–60.8%) (Table 3).

## DISCUSSION

Retrospective review by a single expert of archived images from 1000 randomly selected annual TVS examinations from UKCTOCS, in which both ovaries were visualized and identified as normal by the sonographer, demonstrated that visualization of both ovaries could be confirmed with certainty by the expert in only half of the examinations. In the remaining exams, the expert considered that the sonographer had mistaken some other structure for an ovary, most commonly the bowel (Figure 3). This is the first screening study to undertake such an independent review of ovarian scans identified as normal in postmenopausal women. Our findings suggest that self‐reported VR of the ovaries by a sonographer is not reliable as a QC metric unless confirmed by an independent review, and should be used with caution in the future.

It is generally accepted that the success of any screening program for ovarian cancer using TVS is highly dependent on sonographers detecting any small tumors that might exist in either ovary. Models^13^ estimate that the majority of high‐grade serous ovarian cancers progress to Stage III/IV at a median diameter of about 3 cm. Identifying half of these tumors in Stage I/II at annual screen would require detection of tumors of 1.3 cm in diameter, but to achieve a 50% mortality reduction it would be necessary to detect tumors of 0.5 cm in diameter. Identifying such small tumors is very challenging even for expert sonographers. Therefore, different levels of sonographer skill and experience might explain variation in the outcome between the single‐center Kentucky ovarian cancer ultrasound screening study^6^ and the UKCTOCS study, as well as other largescale multicenter trials (e.g. PLCO) in which it is not feasible for a small group of experts to deliver annual population screening.

We cannot assess the impact on stage shift of the discrepancy between Level‐I sonographers and the expert on ovarian visualization in the ultrasound arm of UKCTOCS^14^. However, all archived examinations preceding ovarian cancer diagnosis in the ultrasound arm of UKCTOCS were reviewed in the course of the trial and collation of these results should provide further insights.

A key quality metric in all ultrasound screening trials is self‐reported VR. In UKCTOCS, a quality monitoring program with regular feedback was in place throughout the trial^2^. It included monitoring of self‐reported VR over a period of 6 months together with other data such as ovarian size and missing/inaccurate information entered into the TMS. In addition, after 2008, UKCTOCS Level‐I sonographers with VR below 60% were subject to targeted training^2^. To what extent these measures might have resulted in some sonographers designating the ovary as ‘seen’ when in doubt is difficult to ascertain.

The use of statistical learning techniques to construct a logistic regression classifier raises the possibility of obtaining independent reliable QC metrics that can be applied at low cost to largescale TVS examinations. We report on a classifier that used ovarian dimensions to identify the ovary, which, however, had a low specificity. It is possible that better performance would have been achieved if morphological features had been included in addition to ovary dimensions.

### Strengths and limitations

We are not aware of any other study reporting on a similar independent review of TVS examinations of archived normal ovarian TVS examinations. Key strengths of our study include the large number of examinations and sonographers from multiple centers reflecting the reality of a population‐based ultrasound screening program, archived images being available for 72% of all annual TVS scans performed, random selection of examinations from those classified as normal and use for the expert review of the exact images that were used by the sonographers to measure the ovaries.

A limitation of the study was the stringent criteria used by the software to identify images, which limited the number of images that could be assessed by the QC classifier. In prospective studies, this could be addressed by the sonographer ‘flagging’ the exact ovarian images during scanning. Another major limitation was that the review was performed by only one expert. Given the known subjectivity of TVS, more robust estimates of expert VR would have been obtained by repeat assessment of random subsets of examinations to assess both intra‐ and interobserver variability. In an audit of seven sonographers reporting high VR in UKCTOCS, there was significant variation in interobserver agreement between eight experts (Stott *et al*., in prep.).

### Other studies

The abovementioned audit performed in 2009 involved a similar review of the images used to measure ovaries from TVS exams performed by UKCTOCS sonographers. In that study, eight experts agreed that visualization of both ovaries could not be confirmed in a significant proportion of exams that had been reported as normal. However, further conclusions about UKCTOCS scanning quality could not be made due to the small number (seven) of sonographers audited and the way they were selected.

### Conclusion

Our results suggest that reliable quality control for TVS cannot be achieved when based solely on ovary visualization data that are self‐reported by sonographers. This is because in almost half the annual TVS examinations performed by UKCTOCS after January 2008 it seems the sonographer had mistakenly measured features like the bowel instead of the ovary showing ovary visualization had not been achieved. Furthermore, as the trial protocol specifies that TVS examinations are unsatisfactory when the ovaries have not been properly visualized (Figure 1), the results suggest that almost half of these examinations were unsatisfactory rather than the 2.5% (2820/113 093) recorded as such. The results highlight the subjective nature of grayscale ultrasound imaging and the importance of operator experience in scanning older postmenopausal women. It is uncertain whether or not this affected the sensitivity and stage at detection in the ultrasound arm of UKCTOCS. However, this study does underline the challenges of delivering largescale TVS screening for ovarian cancer and the need to base its quality management on independent as well as objective QC metrics. In this regard, the classifier produced in this study shows some potential, though further research is needed before it could be used in a TVS quality improvement program.

## Supporting information


**Table S1** Visualization rates (VR) from expert review for ‘match’ and ‘no match’ subsets of the study dataset categorized by visualization definition given in Table 2. The ‘match’ subset contains exams for which the exact images used to measure left and right ovaries can be identified by the software and the ‘no match’ subset contains the remaining exams
