# Supplementary material for: Sonographers' self‐reported visualization of normal postmenopausal ovaries on transvaginal ultrasound is not reliable: results of expert review of archived images from UKCTOCS
Source: Ultrasound Obstet Gynecol. 2018 Mar 7;51(3):401–8. doi: 10.1002/uog.18836 (PMC5888153; doi:10.1002/uog.18836)
Supplement: Supplementary file 1 — Table S1 Visualization rates (VR) from expert review for ‘match’ and ‘no match’ subsets of the study dataset categorized by visualization definition given in Table 2. The ‘match’ subset contains exams for which the exact images used to measure left and right ovaries can be identified by the software and the ‘no match’ subset contains the remaining exams [file UOG-51-401-s001.docx]

| **Visualization of postmenopausal ovaries on static grey scale archived images** | **Image used to measure ovary identified by the software** | | **Image not identified by the software** | |
| --- | --- | --- | --- | --- |
|  | **Count (n=534)** | **VR on expert review (%)** | **Count (n=466)** | **VR on expert review (%)** |
| Both | 280 | 52.4% | 222 | 47.6% |
| Left Only | 64 | 12.0% | 64 | 13.7% |
| Right Only | 86 | 16.1% | 76 | 16.3% |
| None | 104 | 19.5% | 104 | 22.3% |
| **TOTAL** | **534** | **100%** | **466** | **100%** |

Supplementary Table 1: Visualization Rates (VR) from expert review for ‘match’ and ‘no match’ subsets of the study dataset categorised by visualization definition given in Table 2. The ‘match’ subset contains exams for which the exact images used to measure left and right ovary can be identified by the software and the ‘no match’ subset contains the remaining exams.
